# Phase 1 dose de-escalation trial of the endogenous folate [6R]-5,10-methylene tetrahydrofolate in combination with fixed-dose pemetrexed as neoadjuvant therapy in patients with resectable rectal cancer

**DOI:** 10.1007/s10637-015-0272-0

**Published:** 2015-07-21

**Authors:** Bengt Gustavsson, Göran Carlsson, Torbjörn Swartling, Göran Kurlberg, Kristoffer Derwinger, Hillevi Björkqvist, Elisabeth Odin, Fernando Gibson

**Affiliations:** Department of Surgery, University of Gothenburg, Sahlgrenska University Hospital/Östra Institute of Clinical Sciences, Gothenburg, Sweden; PharmaGenesis London, 9 Whitehall, 4th Floor, London, SW1A 2DD UK

**Keywords:** [6R]-5,10-methylene tetrahydrofolate, Endogenous folate, Antifolates, Phase 1 trial, Pemetrexed, Neoadjuvant therapy, Rectal cancer

## Abstract

*Background* Modufolin® ([6R]-5,10-methylene tetrahydrofolate; [6R]-MTHF) is an endogenous biomodulator that is being developed as an alternative to leucovorin, a folate prodrug used in the treatment of colorectal cancer. The objective of this phase 1 dose de-escalation trial was to estimate the minimum tolerated dose of [6R]-MTHF to be used in combination with pemetrexed 500 mg/m^2^ in the neoadjuvant treatment of patients with rectal cancer. *Methods* Adult patients (≥18 years) with resectable rectal adenocarcinoma were allocated to [6R]-MTHF doses of 500, 100, 50, and 10 mg/m^2^ in combination with pemetrexed 500 mg/m^2^. [6R]-MTHF was administered as an intravenous (i.v.) bolus injection 1 week prior to the first dose of pemetrexed and then once weekly for 9 weeks; pemetrexed was administered by i.v. infusion once every 21 days for three cycles. *Results* Twenty-four patients (mean [SD] age, 63.1 [12.9] years) were enrolled in the study. A total of 72 treatment-related adverse events (AEs) were reported, of which the most common were fatigue (*n* = 17; 23.6 %), nausea (*n* = 10; 13.9 %), and diarrhea (*n* = 5; 6.9 %). The incidence of treatment-related AEs by [6R]-MTHF dose level (500, 100, 50, 10 mg/m^2^) was 11.1 % (*n* = 8), 13.9 % (*n* = 10), 45.8 % (*n* = 33), and 29.2 % (*n* = 21), respectively. There were no dose-limiting toxicities, and only two (2.8 %) treatment-related AEs were grade 3 in severity. Of the 11 serious AEs reported, none were considered to be related to [6R]-MTHF treatment. *Conclusions* The results of this phase 1 study indicate that the estimated minimum tolerated dose of [6R]-MTHF was 100 mg/m^2^ once weekly in combination with pemetrexed 500 mg/m^2^. The low toxicity profile of [6R]-MTHF supports its further evaluation as a component of systemic chemotherapy in the management of colon and rectal cancer.

**Electronic supplementary material:**

The online version of this article (doi:10.1007/s10637-015-0272-0) contains supplementary material, which is available to authorized users.

## Introduction

The GLOBOCAN 2012 report ranks colorectal cancer as the third most commonly diagnosed cancer (1.36 million cases, 9.7 %) after lung and breast cancer, and the fourth highest cause of cancer death (694,000 deaths) behind lung, liver, and stomach cancer [1]. In the USA, rectal cancer accounts for approximately 25 % of colorectal cancer cases [2], whereas across Europe, the reported proportion of rectal cancer cases is variable, ranging from 27 to 58 % [3].

In patients with early (T1 or T2) rectal cancers involving less than 40 % of the circumference and without lymphovascular invasion, curative resection is a viable and effective therapeutic option [4]. However, for patients with locally advanced (T3 or T4) rectal cancer, the classical treatment approach consists of combined modality therapy, comprising radiation and chemotherapy in the adjuvant (postoperative) setting [5]. This algorithm was adopted in the 1990s, based on seminal studies which showed that adjuvant chemotherapy based on 5-fluorouracil (5-FU), radiation, or combined chemoradiotherapy (CRT) significantly decreased the rates of local recurrence in patients with locally advanced rectal cancer when compared with resection alone [6–8].

The introduction of total mesorectal excision (TME) reduced the local recurrence rate to approximately 10 %, equivalent to that achieved with conventional surgery with adjuvant CRT [9–12]. These findings raised questions regarding the need for adjuvant CRT and whether neoadjuvant CRT might be superior to adjuvant CRT in improving patient outcomes. Although there is no overall or disease-free survival benefit for neoadjuvant compared with adjuvant CRT [13–16], multiple phase 3 studies have demonstrated that neoadjuvant fluoropyrimidine-based CRT decreases local recurrence rates, induces tumor downstaging, improves the odds of anal sphincter preservation, and increases response rates compared with adjuvant CRT [17–21]. These findings led to the adoption of neoadjuvant CRT followed by TME and adjuvant chemotherapy as the current standard of care for the treatment of locally advanced rectal cancer [22, 23].

**Fig. 1:**
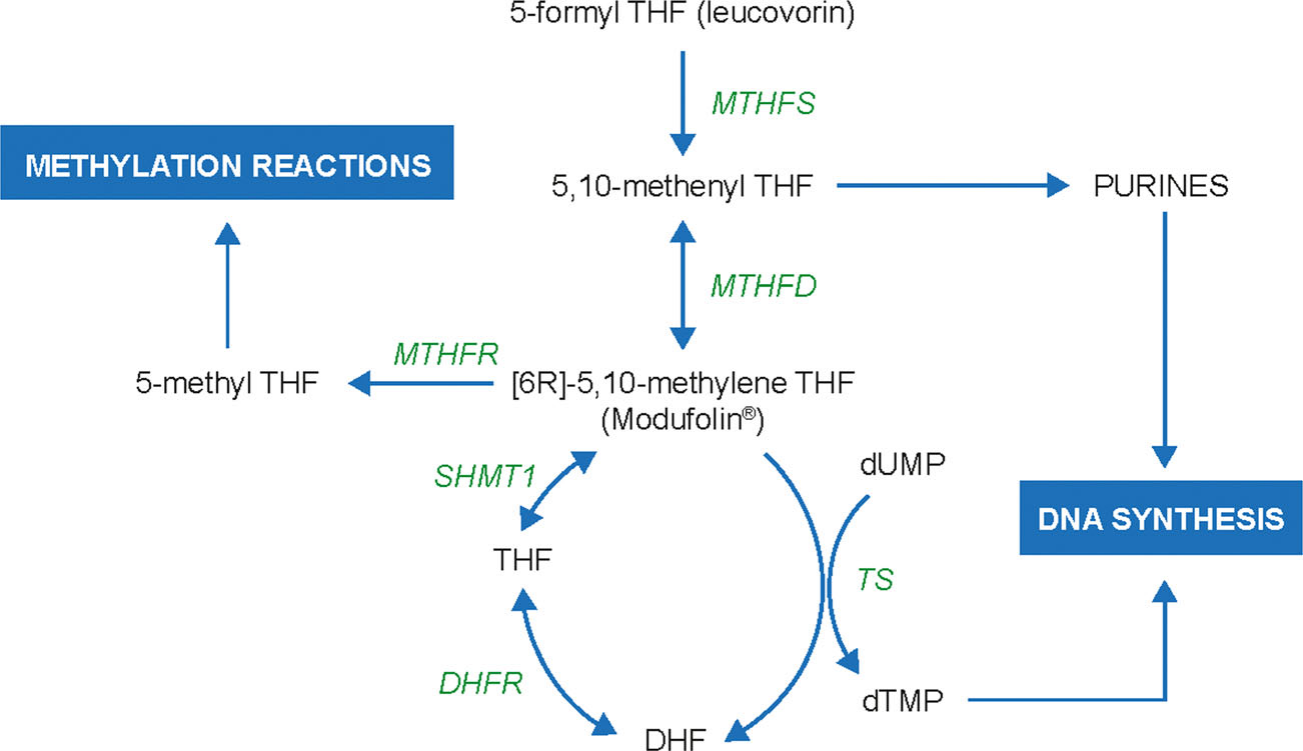
Overview of folate metabolism. *Abbreviations: DHF* dihydrofolate, *DHFR* dihydrofolate reductase, *dTMP* deoxythymidine monophosphate, *dUMP* deoxyuridine monophosphate, *MTHFD* methylene tetrahydrofolate dehydrogenase, *MTHFR* methylene tetrahydrofolate reductase, *MTHFS* methylene tetrahydrofolate synthase, *SHMT1* serine hydroxymethyl transferase 1, *THF* tetrahydrofolate, *TS* thymidylate synthase

Despite the improved local control with neoadjuvant CRT, approximately a third of patients develop distant metastases [24]. With this in mind, strenuous efforts have been made to investigate various combinations and dosing schedules of chemotherapeutic agents with the aim of improving the safety and efficacy of neoadjuvant CRT [25]. The reduced folate leucovorin (5-formyl tetrahydrofolate) potentiates the cytotoxic activity of 5-FU, an inhibitor of thymidylate synthase [26–30], and is an important component of fluoropyrimidine-based chemotherapy in the treatment of colorectal cancer [31, 32]. However, leucovorin is a prodrug that requires metabolic conversion to the active cofactor [6R]-5,10-methylene tetrahydrofolate ([6R]-MTHF; Fig. 1). In the presence of 5-FU, MTHF forms a stable, ternary complex with 5-fluorodeoxyuridine monophosphate and thymidylate synthase, thereby inhibiting thymidylate synthase activity [33–35]. MTHF levels have been shown to be among the lowest of the reduced folates in tumor cells following leucovorin administration [36], suggesting the hypothesis that the direct administration of MTHF might provide superior antitumor activity to that of leucovorin, particularly in patients who convert leucovorin to MTHF at a slow rate or catabolize leucovorin rapidly. Preclinical data in support of this idea was provided by Carlsson et al. [37], who found that the administration of 5-FU in combination with MTHF completely eliminated tumor take in rats inoculated with colon adenocarcinoma cells.

Pemetrexed is a folate analogue that inhibits several folate-dependent enzymes in addition to thymidylate synthase, and was therefore thought to have theoretical advantages over fluoropyrimidines in the treatment of colorectal cancer [38]. Underhill et al. [39] reported the results of a randomized phase 2 trial of pemetrexed plus irinotecan (ALIRI) versus 5-FU plus leucovorin plus irinotecan (FOLFIRI) in patients with advanced or metastatic colorectal cancer. Progression-free survival in the ALIRI arm was significantly shorter than in the FOLFIRI arm (ALIRI, 5.7 months; FOLFIRI, 7.7 months; *p* < 0.001), and the number of drug-related deaths was higher (ALIRI, 4; FOLFIRI, 1), indicating that pemetrexed is not superior to 5-FU-based therapy in the treatment of metastatic colorectal cancer, in terms of either efficacy or safety. However, a phase 1/2 feasibility trial of preoperative pemetrexed therapy in patients with resectable rectal cancer showed a significant reduction of tumor size (*p* < 0.001) and tumor-related symptoms (*p* < 0.018), with a low incidence (10.8 %) of grade 3 or 4 adverse effects [40], indicating that pemetrexed is a promising treatment option in the neoadjuvant setting.

Modufolin® (Isofol Medical AB, Gothenburg, Sweden) is a pure, stable formulation of the [6R]-diastereoisomer of MTHF that was developed as an alternative to leucovorin for the treatment of colon and rectal cancer [41]. Wettergren et al. [42] recently reported the results of a comparative pharmacokinetic/pharmacodynamic study of [6R]-MTHF and leucovorin in patients with colon cancer. The administration of [6R]-MTHF resulted in significantly (*p* < 0.01) higher concentrations of MTHF in mucosa and tumors than were seen following leucovorin administration.

The objective of this phase 1 dose de-escalation trial was to estimate the minimum tolerated dose of [6R]-MTHF to be used in combination with pemetrexed 500 mg/m^2^ in the neoadjuvant treatment of patients with resectable rectal cancer.

## Methods

### Patients

Adult patients (≥18 years of age) with histologically confirmed, resectable adenocarcinoma of the rectum were eligible for inclusion in this single-center, phase 1, open-label, dose de-escalation trial (ISO-MC-091; ClinicalTrials.gov: NCT01397305; EudraCT 2009-009999-12A). The study was conducted between 14 April 2011 and 16 May 2014. Patients were required to have adequate hematologic function (hemoglobin ≥9 g/L, neutrophils ≥1.5 × 10^9^/L, platelet count ≥100 × 10^9^/L), hepatobiliary function (serum bilirubin <1.5 × upper limit of normal range [ULNR], alkaline phosphatase <3 × ULNR, aspartate aminotransferase <3 × ULNR), and renal function (estimated Cockcroft clearance ≥45 mL/min).

Patients were excluded if they had a concurrent, uncontrolled medical illness, or previous or current malignant disease.

Baseline screening evaluations comprised a complete medical examination, including blood cell count and routine blood chemistry, and tumor assessment using radiography. Pre-treatment radiological investigations included a chest X-ray and magnetic resonance imaging (MRI) scans of the upper abdomen (liver) and pelvis. A second MRI scan of the liver and pelvis was performed after completion of the three pemetrexed treatment cycles (as described in the study treatment section below) and before surgery. Staging was performed on pelvic T2-weighted images and a quantitative value of tumor size was measured as the maximal tumor area (mm^2^) on oblique axial T2-weighted images. Post-operative radiological evaluations and tumor outcomes were assessed according to the Response Evaluation Criteria in Solid Tumours (RECIST) criteria [43].

The trial was conducted with local ethical committee approval and in accordance with Good Clinical Practice and the Declaration of Helsinki. All patients provided written informed consent.

### Study treatment and [6R]-MTHF dose de-escalation

Treatment commenced within 4 weeks of the completion of assessments of eligibility. This phase 1 trial utilized a dose de-escalation design to determine the minimum tolerated dose of [6R]-MTHF that is able to abrogate pemetrexed-associated toxicity. In an initial segment of the trial, patients were sequentially assigned to [6R]-MTHF doses of 100, 50, and 10 mg/m^2^, with intrapatient dose de-escalation not permitted. A cohort of six patients was treated at each [6R]-MTHF dose level; however, one patient assigned to receive [6R]-MTHF 10 mg/m^2^ was erroneously given a dose of 50 mg/m^2^. Patients were only treated at the next (lower) [6R]-MTHF dose if ≤2 of the 6 (≤33.3 %) patients reported a dose-limiting toxicity, defined as a grade 3 or 4 drug-related hematologic or gastrointestinal toxicity [44] at the current dose. Following a preliminary analysis of safety data, the study protocol was amended to include an additional [6R]-MTHF dose of 500 mg/m^2^.

[6R]-MTHF was administered as an intravenous (i.v.) bolus injection 1 week prior to the first dose of pemetrexed and then once weekly for 9 weeks; pemetrexed 500 mg/m^2^ was administered by i.v. infusion once every 21 days for three cycles. Concomitant medication included vitamin B12 1000 μg by intramuscular injection 1–2 weeks prior to the first dose of pemetrexed, and thereafter approximately once every 9 weeks, and two dexamethasone 4 mg oral doses on the day before, the day of, and the day after each administration of pemetrexed. Furthermore, if deemed indicated by the treating physician and the multidisciplinary team at the hospital, patients received radiotherapy for 5 consecutive days (at the earliest on day 17 in cycle 3) as standard care before surgery. Treatment was discontinued in the case of disease progression, unacceptable toxicity, or withdrawal of patient consent.

### Toxicity assessment

Adverse events (AEs) were assessed using the Medical Dictionary for Regulatory Activities v11.0 and graded according to the National Cancer Institute Common Terminology Criteria for Adverse Events (NCI CTCAE) v3.0 as toxicity grade 1 (mild), grade 2 (moderate), grade 3 (severe), grade 4 (life-threatening or disabling), or grade 5 (death related to AE) [44]. The relationship of each AE to study drugs and/or procedures was assessed by the investigator. No distinction was made regarding relationship to [6R]-MTHF or pemetrexed unless the AE fulfilled the criteria of a serious AE, which was defined as a life-threatening AE, inpatient hospitalization or prolongation of existing hospitalization, a persistent or significant incapacity or substantial disruption of the ability to conduct normal life functions, or a congenital anomaly/birth defect [45].

## Results

### Patients and dose de-escalation schedule

A total of 24 patients with resectable adenocarcinoma of the rectum (mean age, 63.1 years) were enrolled in the trial and assigned to [6R]-MTHF 500 mg/m^2^ (*n* = 6), 100 mg/m^2^ (*n* = 6), 50 mg/m^2^ (*n* = 7), and 10 mg/m^2^ (*n* = 5). Baseline demographics are summarized in Table 1. The [6R]-MTHF dose de-escalation schedule is shown in Table 2.

**Table 1:** Baseline demographics and disease characteristics

	[6R]-MTHF dose, mg/m^2^				Overall
	500	100	50	10	
Patients, n	6	6	7	5	24
Age, years, mean (SD)	58.17 (8.8)	62.33 (16.0)	68.57 (14.3)	62.40 (11.9)	63.1 (12.9)
Sex, n (%)					
Female	4 (66.7)	3 (50.0)	2 (28.6)	1 (20.0)	10 (41.7)
Male	2 (33.3)	3 (50.0)	5 (71.4)	4 (80.0)	14 (58.3)
Tumor stage (I/II/III/IV)	0/2/4/0	0/1/4/1	0/1/5/0^a^	0/2/3/0	0/6/16/1

*Abbreviations*: *MTHF* 5,10-methylene tetrahydrofolate, *SD* standard deviation

^a^Staging data is missing for one patient in the 50 mg/m2 group because the patient was withdrawn before final staging could be done

**Table 2:** [6R]-MTHF dose de-escalation schedule

[6R]-MTHF dose, mg/m^2^	Patients, n	Doses, n^a^
500	6	60
100	6	60
50	7	70
10	5	50
Total	24	240

*Abbreviation*: *MTHF* 5,10-methylene tetrahydrofolate

^a^All patients received 10 once-weekly doses of [6R]-MTHF, plus pemetrexed 500 mg/m^2^ every 21 days, for three cycles

### Toxicity assessments

Of the 24 enrolled patients, 22 experienced at least one treatment-emergent AE during the study (Table 3). An initial total of 172 treatment-emergent AEs were reported, of which 13 were procedure-related (related to the surgical procedure). After correction for multiple reporting of the same AE code by the same patient, 128 unique treatment-emergent AEs remained. A comprehensive listing of all treatment-emergent AEs is presented in Online Resource 1.

**Table 3:** Overview of adverse events

	[6R]-MTHF dose, mg/m^2^				
	500	100	50	10	Total, n (%)
Patients, n	6	6	7	5	24
Patients with ≥1 treatment-emergent AE, n	4	6	7	5	22
Treatment-emergent AEs, n (%)	24 (18.8)	16 (12.5)	52 (40.6)	36 (28.1)	128 (100)
Patients with ≥1 serious AE, n	1	0	3	1	5
Serious AEs, n (%)^a^	1 (0.8)	0	9 (7.0)	1 (0.8)	11 (8.6)
Patients with ≥1 treatment-related AE, n	2	6	7	5	20
Treatment-related AEs, n (%)	8 (11.1)	10 (13.9)	33 (45.8)	21 (29.2)	72 (100)
Patients discontinued due to AEs, n	0	0	0	0	0
Deaths, n	0	0	0	0	0

All patients received the indicated dose of [6R]-MTHF plus pemetrexed 500 mg/m^2^. Data presented are the number of unique AEs after correction for multiple reporting of of the same AE code by a patient

*Abbreviations*: *AE* adverse event, *MTHF* 5,10-methylene tetrahydrofolate

^a^Percentages based on the total number of treatment-emergent AEs (*n* = 128)

The incidence of treatment-emergent AEs by [6R]-MTHF dose level (500, 100, 50, 10 mg/m^2^) was 18.8 % (*n* = 24), 12.5 % (*n* = 16), 40.6 % (*n* = 52), and 28.1 % (*n* = 36), respectively (Table 3). The most frequently reported treatment-emergent AEs were fatigue (*n* = 18; 14.1 %) and nausea (*n* = 11; 8.6 %); only one of each AE was toxicity grade 3 (Table 4).

**Table 4:** Treatment-emergent adverse events with an incidence of ≥5 %

[6R]-MTHF dose, mg/m^2^	500			100			50			10				
Patients, n	6			6			7			5				
Toxicity grade	1	2	3	1	2	3	1	2	3	1	2	3	4	Total, n (%)
Treatment-emergent AEs, n														
All	17	5	2	14	2		37	10	5	26	8	1	1	128 (100)
Fatigue	2			4			5	1	1	4	1			18 (14.1)
Nausea	2			2			2	2	1		2			11 (8.6)
Pain	2			1				1	1		2			7 (5.5)^a^

Data presented are the number of unique treatment-emergent AEs after correction for multiple reporting of the same AE code by a patient

*Abbreviations*: *AE* adverse event, *MTHF* 5,10-methylene tetrahydrofolate

^a^Aggregate incidence of all pain-related AEs, comprising general pain, abdominal pain, headache, lower leg pain, other leg pain, rectal pain, and shoulder pain

A total of 72 treatment-related AEs were reported by 20 patients (Table 5). The incidence of treatment-related AEs by [6R]-MTHF dose level (500, 100, 50, 10 mg/m^2^) was 11.1 % (*n* = 8), 13.9 % (*n* = 10), 45.8 % (*n* = 33), and 29.2 % (*n* = 21), respectively (Table 3). The most frequent treatment-related AEs were fatigue (*n* = 17; 23.6 %) and nausea (*n* = 10; 13.9 %). Two treatment-related AEs were toxicity grade 3 (Table 5).

**Table 5:** Treatment-related adverse events with an incidence of ≥5 %

[6R]-MTHF dose, mg/m^2^	500			100			50			10			
Patients, n	6			6			7			5			
Toxicity grade	1	2	3	1	2	3	1	2	3	1	2	3	Total, n (%)
Treatment-related AEs, n													
All	8			10			33			21			72 (100)
Fatigue	2			2	2		2	4		4	1		17 (23.6)
Nausea	2			2			2	2			2		10 (13.9)
Diarrhea							2		1		1	1	5 (6.9)

Data presented are the number of unique treatment-related AEs after correction for multiple reporting of the same AE code by a patient

No distinction was made regarding relationship to [6R]-MTHF or pemetrexed unless AEs were assessed as serious AEs

*Abbreviations*: *AE* adverse event, *MTHF* 5,10-methylene tetrahydrofolate

Overall, [6R]-MTHF in combination with pemetrexed was associated with a low toxicity profile. No dose-limiting toxicities were reported during the study, and there were no treatment-related grade 4 or 5 AEs. A total of 11 serious AEs (SAEs) were reported; however, none of these were considered to be related to [6R]-MTHF treatment (Table 3). One SAE (fever) was judged to be related to pemetrexed, four were procedure-related (postoperative wound complication, infection, anastomotic leak, abscess [local anastomosis]), and the rest were considered not to be treatment- or procedure-related. Finally, there were no deaths during the study, and no patients discontinued the study due to AEs or SAEs.

## Discussion

[6R]-MTHF is an enantiomerically pure formulation of the endogenous, biologically active isomer of MTHF [46]. Clinical trials are in progress to evaluate the safety and efficacy of [6R]-MTHF as an alternative to leucovorin in potentiating the activity of 5-FU and the antifolates (including pemetrexed, raltitrexed, pralatrexate, and methotrexate) in the treatment of patients with colon and rectal cancer. Leucovorin is a prodrug that requires metabolic conversion to the active cofactor MTHF [36], and a corollary of this is that leucovorin administration is associated with considerable intra- and interpatient variability in plasma, tumor, and mucosa MTHF levels [47–50]. Thus, we hypothesized that direct administration of MTHF might provide higher intratumor concentrations of MTHF and, therefore, greater antitumor activity than that seen with leucovorin. Indeed, Wettergren et al. [42] recently showed that administration of [6R]-MTHF 200 mg/m^2^ resulted in significantly higher MTHF levels in mucosa and tumor tissue in patients with colon cancer, when compared with administration of leucovorin 200 mg/m^2^. However, it has still to be demonstrated that higher intratumor levels of MTHF translate into superior efficacy in terms of tumor response rates and overall survival.

In the current study, we utilized a dose de-escalation design to identify the optimum dose of [6R]-MTHF to be used in combination with pemetrexed 500 mg/m^2^. The rationale behind the de-escalation design was that [6R]-MTHF was acting as a substitute for folic acid, which is routinely given (together with vitamin B12) to patients receiving pemetrexed in order to minimize the risk of severe toxicity [51–53]. Therefore, a key objective was to identify the minimum tolerated dose of [6R]-MTHF that can be used in combination with pemetrexed 500 mg/m^2^. Although there were no dose-limiting toxicities at any of the four [6R]-MTHF dose levels, the incidence of treatment-related AEs was markedly lower at the two higher [6R]-MTHF doses (500 mg/m^2^, 11.1 %; 100 mg/m^2^, 13.9 %) than at the two lower doses (50 mg/m^2^, 45.8 %, 10 mg/m^2^, 29.2 %), suggesting that [6R]-MTHF was able to modulate the toxicity of pemetrexed in a dose-dependent manner. Given that the incidence of treatment-related AEs was similar at [6R]-MTHF 500 and 100 mg/m^2^, the minimum tolerated [6R]-MTHF dose was estimated to be 100 mg/m^2^ once weekly.

The most frequent treatment-related AEs were grade 1 fatigue, nausea, and diarrhea, which are common side effects of pemetrexed therapy [54]. In contrast to previous trials of pemetrexed in patients with colorectal cancer, none of the patients in the current study reported instances of grade 3/4 neutropenia [39, 40, 55]. However, this was a phase 1 study, and larger phase 2/3 studies will provide a more rigorous evaluation of the safety and tolerability of [6R]-MTHF plus pemetrexed combination therapy. With regard to SAEs, none were considered to be related to [6R]-MTHF, corroborating the toxicity findings of Wettergren et al. [42], who reported that no AEs/SAEs were related to [6R]-MTHF treatment.

## Conclusions

In this phase 1 dose de-escalation trial, [6R]-MTHF doses ranging from 500 mg/m^2^ down to 10 mg/m^2^, in combination with pemetrexed 500 mg/m^2^, showed a low toxicity profile in patients with resectable rectal cancer. The estimated minimum tolerated dose of [6R]-MTHF was 100 mg/m^2^ once weekly. Phase 2 studies are in progress to further assess the safety, tolerability, and efficacy of [6R]-MTHF as a component of systemic chemotherapy in the management of colon and rectal cancer.

## Electronic supplementary material

Online Resource 1 (DOCX 48 kb)
