# Supplementary material for: Phase 1 dose de-escalation trial of the endogenous folate [6R]-5,10-methylene tetrahydrofolate in combination with fixed-dose pemetrexed as neoadjuvant therapy in patients with resectable rectal cancer
Source: Invest New Drugs. 2015 Jul 21;33:1078–85. doi: 10.1007/s10637-015-0272-0 (PMC4768212; doi:10.1007/s10637-015-0272-0)
Supplement: Supplementary file 1 — (DOCX 48 kb) [file 10637_2015_272_MOESM1_ESM.docx]

**Phase 1 dose de-escalation trial of Modufolin^®^ ([6R]-5,10-methylene tetrahydrofolate) in combination with fixed-dose pemetrexed as neoadjuvant therapy in patients with resectable rectal cancer**

B. Gustavsson, G. Carlsson, T. Swartling, G. Kurlberg, K. Derwinger, H. Björkqvist, E. Odin, Department of Surgery, University of Gothenburg, Sahlgrenska University Hospital/Östra Institute of Clinical Sciences, Gothenburg, Sweden

F. Gibson (🖂), PharmaGenesis London, 9 Whitehall, 4th Floor, London, SW1A 2DD, UK; e-mail: [Fernando.Gibson@pharmagenesis.com](mailto:Fernando.Gibson@pharmagenesis.com)

**Online Resource 1** Complete list of treatment-emergent adverse events

| **[6R]-MTHF dose, mg/m^2^** | **500** | | | **100** | | | **50** | | | **10** | | | |  |
| --- | --- | --- | --- | --- | --- | --- | --- | --- | --- | --- | --- | --- | --- | --- |
| **Patients, n** | 6 | | | 6 | | | 7 | | | 5 | | | |  |
| **Toxicity grade** | 1 | 2 | 3 | 1 | 2 | 3 | 1 | 2 | 3 | 1 | 2 | 3 | 4 | **Total, n (%)^a^** |
| **NCI CTCAE:**  **System organ class/**  **lowest level term** |  |  |  |  |  |  |  |  |  |  |  |  |  |  |
| **Blood and lymphatic system disorders** |  |  |  |  |  |  |  |  |  |  |  |  |  |  |
| Anemia | 1 | 2 |  |  |  |  |  |  |  | 1 |  |  |  | 4 (3.1) |
| Hypokalemia |  |  | 1 |  |  |  |  |  |  |  |  |  |  | 1 (0.8) |
| **Cardiac disorders** |  |  |  |  |  |  |  |  |  |  |  |  |  |  |
| Palpitations | 1 |  |  |  |  |  |  |  |  |  |  |  |  | 1 (0.8) |
| **Eye disorders** |  |  |  |  |  |  |  |  |  |  |  |  |  |  |
| Dry eye |  |  |  | 1 |  |  | 1 |  |  | 2 |  |  |  | 4 (3.1) |
| Watering eyes | 1 |  |  |  |  |  | 2 |  |  | 2 |  |  |  | 5 (3.9) |
| **Gastrointestinal disorders** |  |  |  |  |  |  |  |  |  |  |  |  |  |  |
| Abdominal distension |  |  |  |  |  |  |  | 1 |  | 1 |  |  |  | 2 (1.6) |
| Abdominal pain |  |  |  |  |  |  |  | 1 |  |  |  |  |  | 1 (0.8) |
| Constipation |  |  |  |  | 1 |  | 1 |  |  | 1 |  |  |  | 3 (2.3) |
| Diarrhea |  |  |  |  |  |  | 2 |  | 2 |  | 1 | 1 |  | 6 (4.7) |
| Dyspepsia |  |  |  |  |  |  | 1 |  |  | 1 |  |  |  | 2 (1.6) |
| Flatulence | 1 |  |  |  |  |  | 1 |  |  | 1 |  |  |  | 3 (2.3) |
| Rectal leak |  |  |  |  |  |  |  |  |  |  | 1 |  |  | 1 (0.8) |
| Sense of heat in abdomen | 1 |  |  |  |  |  |  |  |  |  |  |  |  | 1 (0.8) |
| Gastrointestinal pain | 1 |  |  | 1 |  |  | 2 |  |  |  |  |  |  | 4 (3.1) |
| Mucositis oral |  |  |  |  |  |  | 1 |  |  | 1 |  |  |  | 2 (1.6) |
| Nausea | 2 |  |  | 2 |  |  | 1 | 2 | 1 |  | 2 |  |  | 10 (7.8) |
| Obstipation |  |  |  |  |  |  | 1 |  |  |  |  |  |  | 1 (0.8) |
| Rectal pain |  |  |  |  |  |  |  |  | 1 |  | 1 |  |  | 2 (1.6) |
| Vomiting |  |  |  |  | 2 |  |  |  |  |  |  |  |  | 2 (1.6) |
| **General disorders and administration site conditions** |  |  |  |  |  |  |  |  |  |  |  |  |  |  |
| Loss of apetite |  |  |  |  |  |  | 1 |  |  |  |  |  |  | 1 (0.8) |
| Swollen lips |  |  |  |  |  |  | 1 |  |  |  |  |  |  | 1 (0.8) |
| Pain |  |  |  |  |  |  |  |  |  |  | 1 |  |  | 1 (0.8) |
| Fatigue | 2 |  |  | 4 |  |  | 5 | 1 | 1 | 4 | 1 |  |  | 18 (14.1) |
| Fever |  |  |  |  |  |  | 1 |  |  | 1 |  |  |  | 2 (1.6) |
| Flu-like symptoms | 1 |  |  |  |  |  |  |  |  |  |  |  |  | 1 (0.8) |
| Gait disturbance |  |  |  |  |  |  | 1 |  |  |  |  |  |  | 1 (0.8) |
| **Infections and infestations** |  |  |  |  |  |  |  |  |  |  |  |  |  |  |
| Anorectal infection |  |  | 1 |  |  |  |  |  |  |  |  |  |  | 1 (0.8) |
| Herpes zoster |  |  |  |  |  |  |  | 1 |  |  |  |  |  | 1 (0.8) |
| Virus |  | 1 |  |  |  |  |  |  |  |  |  |  |  | 1 (0.8) |
| Upper respiratory infection |  |  |  | 1 |  |  |  |  |  |  |  |  |  | 1 (0.8) |
| Wound infection |  |  |  |  |  |  |  | 1 |  |  |  |  | 1 | 2 (1.6) |
| **Investigations** |  |  |  |  |  |  |  |  |  |  |  |  |  |  |
| Weight gain |  |  |  |  |  |  | 1 |  |  |  |  |  |  | 1 (0.8) |
| **Metabolism and nutrition disorders** |  |  |  |  |  |  |  |  |  |  |  |  |  |  |
| Anorexia |  |  |  |  |  |  | 2 |  |  |  |  |  |  | 2 (1.6) |
| **Musculoskeletal and connective tissue disorders** |  |  |  |  |  |  |  |  |  |  |  |  |  |  |
| Shoulder pain |  |  |  | 1 |  |  |  |  |  |  |  |  |  | 1 (0.8) |
| Pain in the extremity | 1 |  |  |  |  |  |  |  |  | 1 |  |  |  | 2 (1.6) |
| **Nervous system disorders** |  |  |  |  |  |  |  |  |  |  |  |  |  |  |
| Cognitive disturbance |  |  |  | 1 |  |  |  |  |  |  |  |  |  | 1 (0.8) |
| Dizziness | 1 | 1 |  |  |  |  | 1 |  |  |  |  |  |  | 3 (2.3) |
| Dysgeusia | 1 |  |  |  |  |  | 2 |  |  | 2 |  |  |  | 5 (3.9) |
| Headache | 1 |  |  |  |  |  |  |  |  | 1 |  |  |  | 2 (1.6) |
| Neuralgia |  |  |  | 1 |  |  |  |  |  |  |  |  |  | 1 (0.8) |
| **Psychiatric disorders** |  |  |  |  |  |  |  |  |  |  |  |  |  |  |
| Agitation |  |  |  |  |  |  | 1 |  |  |  |  |  |  | 1 (0.8) |
| Anxiety |  |  |  |  |  |  |  | 1 |  | 1 |  |  |  | 2 (1.6) |
| Depression | 1 |  |  |  |  |  |  |  |  |  |  |  |  | 1 (0.8) |
| Insomnia | 1 |  |  | 1 | 1 |  | 1 |  |  | 1 |  |  |  | 5 (3.9) |
| Feeling of discomfort | 1 |  |  |  |  |  |  |  |  |  |  |  |  | 1 (0.8) |
| **Renal and urinary disorders** |  |  |  |  |  |  |  |  |  |  |  |  |  |  |
| Urinary frequency |  |  |  |  |  |  |  |  |  | 1 |  |  |  | 1 (0.8) |
| **Respiratory, thoracic and mediastinal disorders** |  |  |  |  |  |  |  |  |  |  |  |  |  |  |
| Cough |  |  |  |  |  |  |  |  |  |  | 1 |  |  | 1 (0.8) |
| Epistaxis |  |  |  |  |  |  |  |  |  | 2 |  |  |  | 2 (1.6) |
| Dripping nose |  |  |  |  |  |  | 1 |  |  |  |  |  |  | 1 (0.8) |
| **Skin and subcutaneous tissue disorders** |  |  |  |  |  |  |  |  |  |  |  |  |  |  |
| Dry skin |  |  |  |  |  |  |  |  |  | 1 |  |  |  | 1 (0.8) |
| Hair loss/alopecia |  |  |  | 1 |  |  |  |  |  |  |  |  |  | 1 (0.8) |
| Pruritus |  |  |  |  |  |  |  |  |  | 1 |  |  |  | 1 (0.8) |
| Rash acneiform |  |  |  | 1 |  |  | 2 |  |  | 1 |  |  |  | 4 (3.1) |
| Rash |  |  |  |  |  |  | 1 |  |  |  |  |  |  | 1 (0.8) |
| **Vascular disorders** |  |  |  |  |  |  |  |  |  |  |  |  |  |  |
| Hypertension |  | 1 |  |  |  |  |  |  |  |  |  |  |  | 1 (0.8) |
| **Grade total** | 17 | 5 | 2 | 14 | 2 | 0 | 37 | 10 | 5 | 26 | 8 | 1 | 1 |  |
| **Dose total** | 24 |  |  | 16 |  |  | 52 |  |  | 36 |  |  |  | 128 |

Abbreviations: *AE* adverse event; *MTHF* 5,10-methylene tetrahydrofolate; NCI CTCAE, National Cancer Institute Common Terminology Criteria for Adverse Events

All patients received pemetrexed 500 mg/m^2^ plus the indicated dose of [6R]-MTHF

Data presented are the number of unique treatment-emergent AEs after correction for multiple reporting of of the same AE code by a patient

No distinction was made regarding relationship to [6R]-MTHF or pemetrexed unless the AE was assessed as a serious AE
